# Standards of Care to Practice: Redefining Type 1 Diabetes Care within Learning Health Systems

**DOI:** 10.1097/pq9.0000000000000883

**Published:** 2026-05-18

**Authors:** Melissa Leonard, Manmohan K. Kamboj, Osagie Ebekozien

**Affiliations:** From the * Health Access and Quality Division, The American Diabetes Association, Arlington, Va; † Division of Endocrinology, Nationwide Children’s Hospital, The Ohio State University College of Medicine, Columbus, Ohio.

## INTRODUCTION

For more than 35 years, the American Diabetes Association (ADA) has published annual guidelines for diabetes management in a comprehensive document called the *Standards of Care in Diabetes (Standards of Care*).^1^ The *Standards of Care* include recommendations specific to pediatric type 1 diabetes (T1D). Unfortunately, despite more than 35 years of ADA guidelines, the adoption remains suboptimal. Recent real-world data demonstrate that less than 30% of patients living with T1D are on recommended insulin delivery technology. Less than 25% meet recommended HbA1c targets.^2^ This commentary explores the growing role of quality improvement (QI) and the learning health system (LHS) framework in promoting the faster adoption of national T1D standards of care into routine practice. The ADA framework builds on established, well-published frameworks for LHSs that emphasize using real-time data and evidence to inform processes and foster a culture of continuous learning and improvement.^3^ This process turns knowledge into action, effectively narrowing the gap between research and everyday practice.

### The Need for Early Intervention and Equitable Access to Care for Pediatric T1D

**Fig. 1.**
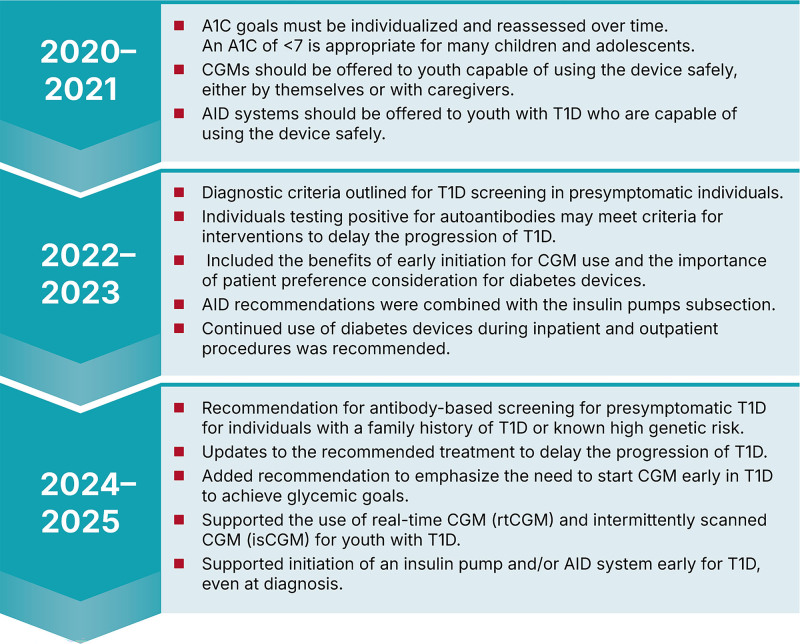
Selected key pediatric T1D recommendation updates (ADA Standards of Care 2020–2025). AID, automated insulin delivery; CGM, continuous glucose monitoring.

T1D is a chronic autoimmune condition characterized by T cell–mediated destruction of pancreatic beta cells, necessitating lifelong insulin treatment.^4^ The prevalence of T1D is steadily increasing worldwide, with cases in North America expected to double by 2040 due to better detection and unknown triggers.^5^ Within the past 5 years, there have been several major updates to the *Standards of Care* recommendations for the screening and management of T1D for children and adolescents. The 2020–2025 evolution of the ADA’s *Standards of Care* includes updates that emphasize earlier screening and evidence-based intervention to slow T1D progression, individualized glycemic goals, and expanded use of diabetes technology (Fig. 1).

There are 3 stages in the development of T1D: stage 1, presymptomatic autoimmunity; stage 2, early diabetes; and stage 3, overt diabetes, characterized by symptomatic clinical manifestations.^6^ The ADA’s *Standards of Care* recommendations have profound implications for early detection and evidence-based intervention aligned with recent scientific advances, such as recommended screening for autoantibodies before symptomatic presentation for those with an elevated risk and early intervention with evidence-based treatments to delay the development of symptomatic T1D.^7^ Screening for T1D in pediatric settings is necessary to detect T1D before symptoms appear, provide treatments to delay the progression of T1D, and mitigate the risk of life-threatening complications such as diabetic ketoacidosis. The ADA also recommends immunotherapy for individuals with stage 2 T1D, highlighting a growing emphasis on early intervention and preventive care.^7^ However, implementation of these standards continues to be a challenge because of cost and staffing limitations, as well as provider readiness, as highlighted in a recent mixed-methods study.^2^

In addition to emphasizing early identification and intervention, the ADA’s 2025 Standards of Care strongly promote the early and sustained use of diabetes technology, including continuous glucose monitoring and automated insulin delivery systems.^7^ The use of these technologies improves glycemic outcomes and should be recommended to all individuals with T1D according to ADA Standards of Care.^7^ Several studies have demonstrated that provider biases can limit the offering of diabetes technology to patients.^8^ The ADA emphasizes the importance of shared decision-making to determine which diabetes technology option works best for a patient’s individual circumstances to increase adherence.^7^ Recent studies have shown that early initiation of diabetes technology can have a significant impact on improving health outcomes.^7^ However, equitable access to diabetes technology remains a barrier, and disparities persist.^8^ The ADA strongly recommends offering diabetes technology to all patients with T1D as early as possible to address this known disparity.^7^

### LHSs: Bridging the Gap Between Evidence and Practice

LHSs are foundational to the ADA’s strategy to improve early intervention, evidence-based treatment, and equitable access to care. These health systems use internal data and experience, along with external evidence, to drive continuous improvement.^3^ Within the LHS framework, providers and clinical teams connect with peers to learn and share experiences, which will improve their own practices and outcomes.^9^ This framework can be used to address systemic barriers, foster inclusive care, and bridge the gap between evidence and application.^10^ Several LHS QI initiatives have yielded measurable improvements in T1D care delivery, management, and outcomes for children and adolescents.^11^

**Fig. 2.**
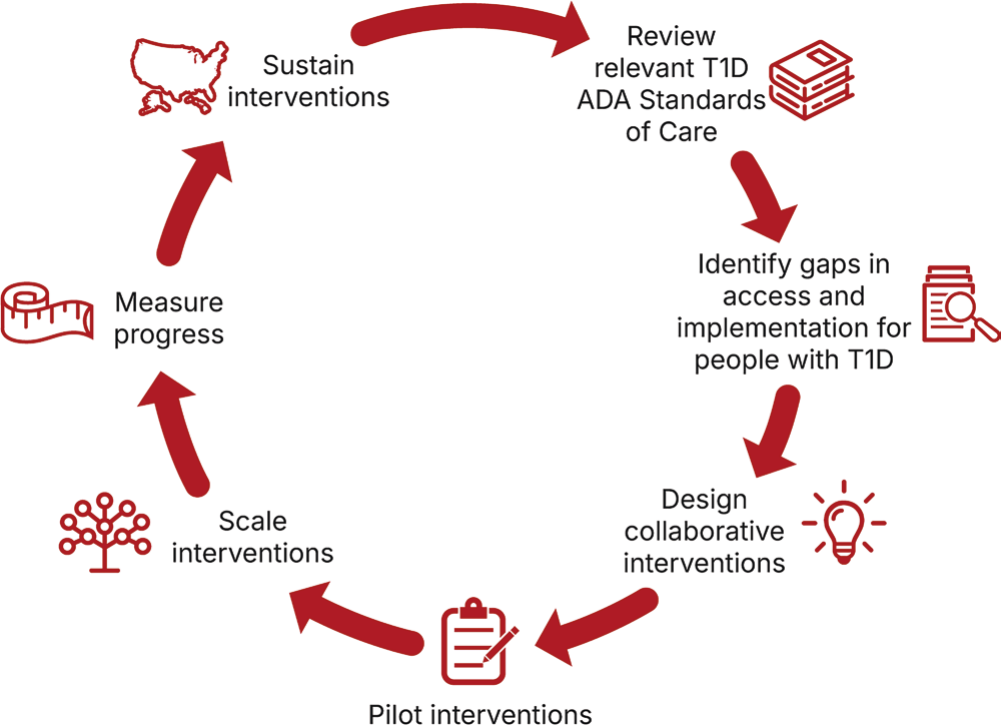
The ADA’s LHS framework for promoting the translation of national T1D Standards of Care to routine standards of practice.

Recognizing that specialty care alone cannot meet the growing need for T1D management, the ADA is using the LHS framework and actively expanding its reach into primary care and community settings. The ADA’s Primary Care Council includes all major primary care professional organizations and actively collaborates on national diabetes efforts. Additionally, the ADA’s Primary Care Alliance, which includes more than 4,000 primary care sites nationwide, focuses on improving outcomes through QI and collaborates with the ADA on QI initiatives that test scalable interventions to improve T1D management in pediatric settings. The LHS framework, along with QI and implementation science methodologies, is used to facilitate data-driven improvement for T1D management within primary care settings and move from standards of care to standards of practice (Fig. 2). The ADA’s quality key drivers include enhancing provider competencies, integrating clinical decision-support tools, and fostering patient engagement. This structure provides the support that health systems need to quickly and effectively integrate rapidly evolving recommendations due to continuous scientific advancements in T1D identification and management.

The ADA’s *Standards of Care* explicitly recognize the linkage between equity and quality. The ADA’s standards emphasize the need to address disparities by encouraging the universal provision of diabetes technology to all individuals with T1D and by recommending stratifying clinical data by race and ethnicity, insurance status, language, and other social determinants of health.^7,12^ Additionally, embedding equity into QI strategies is encouraged to ensure equity-focused quality goals.^13^ The ADA’s standards also highlight the importance of identifying social determinants of health needs and the connection to community resources, along with the universal offering of diabetes technology.^7^ QI tools can be repurposed to address biases and enhance equity in clinical practices.^14^ This shift to focus on equity is essential for addressing health disparities and improving access to care for all children and adolescents with T1D.

## CONCLUSIONS

The ADA’s *Standards of Care* have rapidly evolved to provide evidence-based, comprehensive recommendations for high-quality care for children and adolescents living with T1D. QI and LHSs are needed to facilitate the efficient and effective implementation of these standards into routine standards of practice. Furthermore, integrating social support and culturally tailored interventions must be prioritized to ensure equitable and inclusive care. The ADA’s facilitation of the transition from the standards of care to standards of practice represents a call to action. By leveraging LHSs, prioritizing early intervention with evidence-based treatments, and embedding equity into every facet of care, the ADA is redefining T1D management in children and adolescents. As clinical teams balance the complexities of the healthcare system with patient needs, the ADA’s approach provides a compelling case for data-driven, equitable, person-centered care.
